# Overlap time is an independent risk factor of radiation pneumonitis for patients treated with simultaneous EGFR-TKI and thoracic radiotherapy

**DOI:** 10.1186/s13014-021-01765-x

**Published:** 2021-02-23

**Authors:** Wenxiao Jia, Qianqian Gao, Min Wang, Ji Li, Wang Jing, Jinming Yu, Hui Zhu

**Affiliations:** 1 Department of Radiation Oncology, Shandong Cancer Hospital and Institute Affiliated to Shandong University, Shandong First Medical University, Shandong Academy of Medical Sciences, 440 Jiyan Road, Jinan, 250117 Shandong Province China; 2 Department of Obstetrics and Gynecology, Qilu Hospital Affiliated to Shandong University, Jinan, 250012 Shandong Province China; 3 Department of Radiation Oncology, Shandong Cancer Hospital and Institute Affiliated to Shandong First Medical University, Shandong Academy of Medical Sciences, Jinan, 250117 Shandong Province China

**Keywords:** Radiation pneumonitis, EGFR-TKI, Thoracic radiotherapy, Non-small cell lung cancer, Risk factor

## Abstract

**Background:**

The exact rate and relevant risk factors of radiation pneumonitis (RP) for non-small-cell cancer (NSCLC) patients treated with the combination of epidermal growth factor receptor-tyrosine kinase inhibitors (EGFR-TKIs) and thoracic radiotherapy have not been reported. Thus, this study aimed to investigate the rate and risk factors of RP for EGFR-positive NSCLC patients simultaneously treated with first-generation EGFR-TKI and TRT.

**Patients and methods:**

We retrospectively evaluated NSCLC patients simultaneously treated with first-generation EGFR-TKI and thoracic radiotherapy between January 2012 and December 2019 at Shandong Cancer Hospital and Institute, Shandong, China. RP was diagnosed via computed tomography and was classified according to the Common Terminology Criteria for Adverse Events v5.0. The risk factors of RP were identified using uni- and multivariate analyses.

**Results:**

Of the 67 patients included, 44.78% (30/67) developed grade ≥ 2 RP. Grade ≥ 2 RP occurred within a median of 3.48 (range 1.07–13.6) months. The EGFR-TKI icotinib, ipsilateral lung V30 > 34%, and overlap time of > 20 days between EGFR-TKI and thoracic radiotherapy were identified to be independent predictive factors of grade ≥ 2 RP.

**Conclusions:**

Grade ≥ 2 RP is highly frequent in NSCLC patients simultaneous treated with first-generation EGFR-TKI and thoracic radiotherapy. Icotinib, ipsilateral lung V30 ≤ 34%, and overlap time of ≤ 20 days for EGFR-TKI and thoracic radiotherapy will be helpful to lower the risk of RP in these patients. The addition of thoracic radiotherapy should be cautious, and the treatment strategies can be optimized to reduce the rate of RP for patients treat with simultaneous EGFR-TKI and thoracic radiotherapy.

## Background

Epidermal growth factor receptor tyrosine kinase inhibitors (EGFR-TKIs) are the standard treatment modality for stage IV NSCLC patients harboring EGFR-sensitive mutations [1, 2]. Among these patients, those who progress despite achieving stable disease during treatment are indicated for thoracic radiotherapy (TRT) to improve local control and prognosis. In 2018, Xu et al. found that the addition of consolidative local ablation treatment (LAT) for stage IV EGFR-mutant oligometastatic NSCLC treated with TKI can significantly improve overall survival (40.5 versus 31.5 months, *P* < 0.001) [3]. This was confirmed in subsequent studies [4–7].

However, despite the survival benefit of the combination of radiotherapy and TKI, the occurrence of radiation pneumonitis (RP) cannot be ignored. In 2014, Zhuang et al. reported that 37.5% (9/24) of patients treated with the combination of erlotinib and TRT experience grade ≥ 2 RP [8]. In our previous study, 63.6% (7/11) of patients simultaneously treated with TRT and osimertinib, developed grade ≥ 2 RP [9]. However, despite this high frequency of RP, its exact rate and risk factors among NSCLC patients treated with the combination of EGFR-TKI and TRT have not been completely evaluated. Thus, this study aimed to explore the rate and risk factors of RP for EGFR-mutant NSCLC patients simultaneously treated with first-generation EGFR-TKI and TRT.

## Patients and methods

### Study design and patients

This was a retrospective study of NSCLC patients who were simultaneously treated with EGFR-TKI and TRT at Shandong Cancer Hospital and Institute between January 2012 and December 2019 were retrieved. The inclusion criteria were as follows: (1) stage IIIB-IVB EGFR-mutant NSCLC and (2) first-generation EGFR-TKI was used (gefitinib, erlotinib, and icotinib). Patients with a history of TRT or interstitial lung disease or those treated with immune checkpoint inhibitors were excluded. Simultaneous treatment was defined as at least one day overlap existed between EGFR-TKI and TRT. The overlap time was the days when TRT and EGFR-TKI were conducted simultaneously, and was affected by the duration of TRT, the timing of EGFR-TKI or TRT was added. Data were collected from the medical records. This study was approved by the institutional review board of Shandong Cancer Hospital and Institute and was performed in accordance with the Declaration of Helsinki.

### Treatment protocol

All patients were treated with intensity-modulated radiotherapy and 3-dimensional conformal radiation therapy with photon therapy. Lung dosimetric parameters were extracted from the treatment planning system. Total lung V5 was defined as the percentage of the total lung volume receiving 5 Gy of radiation. Ipsilateral lung V5 was defined as the percentage of the affected lung volume receiving 5 Gy of radiation. EGFR-TKI treatment comprised gefitinib, erlotinib, or icotinib at 250 mg daily, 150 mg daily, or 125 mg three times daily, respectively.

### Diagnosis and classification of radiation pneumonitis

RP was diagnosed based on patients’ symptom, laboratory tests and imaging findings obtained via computed tomography (CT). Follow-up CT images were independently evaluated by two senior radiologists. Differences were resolved by consulting with a third senior radiologist. RP was graded from 1 to 5 according to the Common Terminology Criteria for Adverse Events v5.0 [10]. The grade 1 RP is asymptomatic, only pneumonitis within radiation field and intervention are not indicated; grade 2 RP present the clinical symptoms such as cough with or without expectoration, chest distress, and breathlessness and so on, their activities of daily living (ADL) is limiting mild, the corticosteroids and some symptomatic treatments are administered; the patient with grade 3 RP have severe symptoms, the selfcare ADL is limited, oxygen indicated; patient with grade 4 RP have life-threatening respiratory compromise, urgent intervention indicated; patient die from grade 5 RP. Clinical symptoms, laboratory tests, treatments, and patient outcomes were included to assess the grade of RP.

### Statistical analysis

Univariate and multivariate analyses using binary logistics regression were conducted to analyze the risk factors of grade ≥ 2 RP. Categorical variables were included directly in the univariate analysis. Meanwhile, for noncategorical variables, receiver operating characteristic (ROC) curves were first generated to determine the optimal cut-off value. The cut-off value was determined according to the maximum Youden index after considering sensitivity and specificity. Then, the continuous variables were converted to categorical variables. Significant variables in the univariate analysis (i.e., those with a *P* value of ≤ 0.05) were included in the multivariate analysis. For variables with collinearity, the those with a smaller *P* value were chosen for multivariate analysis. All statistical analyses were performed using SPSS V26.0 (IBM Corporation, Armonk, NY, USA). A *P* value of ≤ 0.05 was considered statistically significant.

## Results

### Patient characteristics

In total, 67 patients were included in the analysis. The clinical characteristics and relevant dosimetric parameters are presented in Table 1. The median patient age was 53 years (range 37–79 years). The majority of the patients were female (59.70%) and had stage IV disease (80.60%). EGFR exon 19 deletion (44.78%) and EGFR exon 21 L858R (43.28%) were the main mutation types. The median follow-up time was 15.27 months. TRT was performed to improve local control in 16 (23.88%) patients who developed progressive disease (PD) of the primary lesion during EGFR-TKI treatment. Meanwhile, TRT was added for consolidation of the primary lesion in the remaining patients without PD. First-line treatment comprised concurrent first-generation EGFR-TKI and TRT in 16 (23.88%) patients and concurrent first-generation EGFR-TKI, chemotherapy, and TRT in 18 (26.87%) patients. The 33 (49.25%) patients with previous chemotherapy were treated with simultaneous first-generation EGFR-TKI and TRT as second-line treatment.

**Table 1:** Clinicodemographic patient characteristics

Factor	N (%)
Gender (n)	
Male	27 (40.30%)
Female	40 (59.70%)
Age (median, range) (y)	53.01 (36.73–78.35)
Smoking history (n)	
No	50 (74.63%)
Yes	17 (25.37%)
T stage in naïve (n)	
1–2	43 (64.18%)
3–4	24 (35.82%)
TNM stage (n)	
IIIB-IIIC	13 (19.40%)
IVA-IVB	54 (80.60%)
Location (n)	
Upper lobe	21 (31.34%)
Middle and lower lobe	46 (68.66%)
Type of mutation (n)	
EGFR exon 19 del	30 (44.78%)
EGFR exon 21 L858R	29 (43.28%)
Others	8 (11.94%)
Type of EGFR-TKI (n)	
Gefitinib	39 (58.21%)
Erlotinib	17 (25.37%)
Icotinib	11 (16.42%)
The treatment model (n)	
Simultaneous EGFR-TKI and TRT for first line	16 (23.88%)
Simultaneous EGFR-TKI, chemotherapy and TRT for first line	18 (26.87%)
Previous chemotherapy, simultaneous EGFR-TKI and TRT for second line	33 (49.25%)
Dose fractionation (n)	
CFRT	59 (88.06%)
SBRT	8 (11.94%)
Dose per fraction (median, range) (Gy)	2 (2–12)
Total dose (median, range) (Gy)	56 (39–72)
GTV (median, range) (ml)	25.30 (0.70–338.80)
PTV (median, range) (ml)	133.30 (11.40–752.20)

*EGFR-TKI* epidermal growth factor receptor tyrosine kinase inhibitor, *TRT* thoracic radiotherapy, *CFRT* conventional fractional radiotherapy, *SBRT* stereotactic body radiation therapy, *GTV* gross tumor volume, *PTV* plan tumor volume, *RP* radiation pneumonitis

### Incidence of RP

**Fig. 1:**
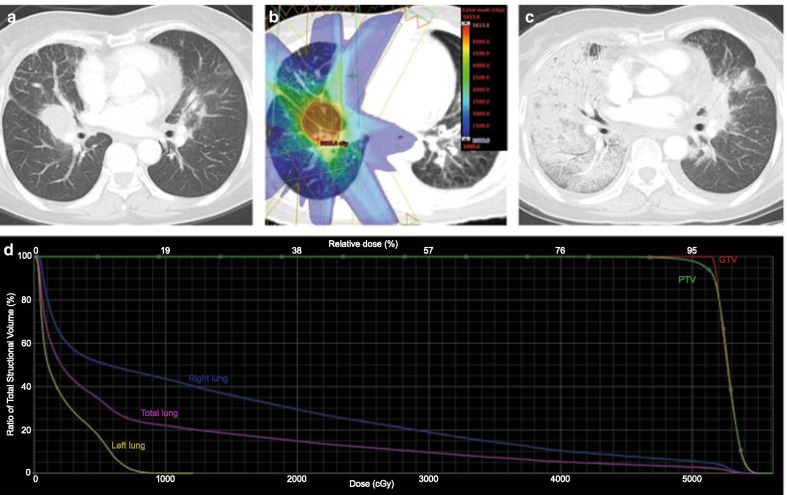
Representative images of one patient who experienced grade 3 RP after simultaneous first-generation EGFR-TKI and thoracic radiotherapy. **a** Primary lesion before radiotherapy. **b** Isodose curve of the treatment plan. **c** Three months after radiotherapy. **d** Dose distribution histogram of the total lung, right lung, and left lung

Overall, 30 of the 67 (44.78%) patients developed grade ≥ 2 RP. Among them, 24 (35.82%) patients and 6 (8.96%) patients had grades 2 and 3 RP, respectively. No patient developed grade 4 or 5 RP. The median time from the beginning of TRT to the occurrence of grade ≥ 2 RP was 3.48 months (range, 1.07–13.6 months). There were 96.67% (29/30) of the patients who developed grade ≥ 2 RP within 6 months of the beginning of TRT. Only one patient experienced grade ≥ 2 RP at a later period of 13.6 months. Representative CT images of one patient who developed grade 3 RP are presented in Fig. 1. The patient received a total of 50 Gy in 10 fractions, and the total mean lung dose (MLD), total lung V20, and ipsilateral lung V30 were 8.21 Gy, 14.91%, and 19.05%, respectively.

### Risk factors of RP

#### Univariate analysis

As shown in Table 2, the overall risk of grade ≥ 2 RP increased as the lung irradiation dose increased. The total lung V15, V20, V30 and ipsilateral lung V15, V20, and V30 MLD were significantly correlated with grade ≥ 2 RP. In view of the collinearity of lung radiation dosimetric parameters, the ipsilateral lung V30 was converted as a categorical variable, and the variable with the smallest *P* value was included in the multivariate analysis.

**Table 2:** Univariate logistic regression analysis of the risk factors of grade ≥ 2 radiation pneumonitis

Factor	*P* value	OR (95% CI)
Total lung V5	0.188	1.02 (0.99–1.06)
Total lung V10	0.069	1.02 (1.00–1.09)
Total lung V15	**0.040**	1.06 (1.00–1.13)
Total lung V20	**0.036**	1.08 (1.01–1.16)
Total lung V30	**0.032**	1.11 (1.01–1.22)
Total lung MLD	0.117	1.11 (0.98–1.25)
Ipsilateral lung V5	0.090	1.02 (1.00–1.05)
Ipsilateral lung V10	0.054	1.03 (1.00–1.06)
Ipsilateral lung V15	**0.021**	1.04 (1.01–1.08)
Ipsilateral lung V20	**0.011**	1.06 (1.01–1.10)
Ipsilateral lung V30	**0.009**	1.07 (1.02–1.13)
Ipsilateral lung MLD	**0.035**	1.09 (1.01–1.17)

The bold text was some risk factor of RP, which were significantly correlated with RP and the *p*-value < 0.05

*MLD* mean lung dose, *RP* radiation pneumonitis

Aside from the lung radiation dosimetric parameters, other clinical characteristics were also included in the univariate analysis. The results are presented in Table 3. The type of mutation, use of chemotherapy, and timing for the addition of TRT were not associated with RP. Meanwhile, a gross tumor volume (GTV) of > 27 ml (OR 2.77; 95% CI 1.02–7.49, *P* = 0.045), PTV > 120 ml (OR 4.03; 95% CI 1.42–11.43; *P* = 0.009), and ipsilateral lung V30 > 34% (OR 7.50; 95% CI 1.48–38.08; *P* = 0.015) were significantly correlated with grade ≥ 2 RP. Compared with gefitinib, icotinib was associated with a lower risk of grade ≥ 2 RP (OR 0.09; 95% CI 0.01–0.74; *P* = 0.025). Similar results were obtained for erlotinib versus icotinib. A dose per fraction of ≥ 3 Gy was also correlated with a lower risk of grade ≥ 2 PR (OR 0.29; 95% CI 0.09–0.94; *P* = 0.039). Further, an overlap time of > 20 days between first-generation EGFR-TKI and TRT and a TRT duration of > 33 days were significantly correlated with grade ≥ 2 RP (OR 8.52; 95% CI 1.75–41.41; *P* = 0.008 and OR 3.05; 95% CI 1.01–9.21; *P* = 0.048, respectively).

**Table 3:** Univariate and multivariate logistic regression analyses of the risk factors of grade ≥ 2 radiation pneumonitis

Factor	Univariate analysis		Multivariate analysis	
	*P* value	OR (95% CI)	*P* value	OR (95% CI)
Gender				
Male				
Female	0.649	0.80 (0.30–2.12)		
Age (years)				
≤ 65				
> 65	0.146	0.42 (0.13–1.36)		
T stage in naïve				
T1–2				
T3–4	0.099	2.36 (0.85–6.55)		
Type of mutation				
Exon 19-del	0.080			
Exon 21 L858R	0.141	0.45 (0.16–1.3)		
Others	0.219	3.00 (0.52–17.32)		
Type of EGFR-TKI				
Gefitinib	0.081		0.081	
Erlotinib	0.641	0.76 (0.24–2.39)	0.808	1.18 (0.32–4.37)
Icotinib	**0.025**	0.09 (0.01–0.74)	**0.030**	0.08 (0.007–0.78)
The treatment model				
Simultaneous EGFR-TKI and TRT for first line	0.448			
Simultaneous EGFR-TKI, chemotherapy and TRT for first line	0.384	1.76 (0.49–6.34)		
Previous chemotherapy, simultaneous EGFR-TKI and TRT for second line	0.206	2.50 (0.60–10.34)		
Dose fractionation				
CFRT				
SBRT	0.558	1.70 (0.29–9.97)		
GTV (ml)				
≤ 27				
> 27	**0.045**	2.77 (1.02–7.49)		
PTV (ml)				
≤ 120				
> 120	**0.009**	4.03 (1.42–11.43)		
Dose per fraction (Gy)				
< 3.00				
≥ 3.00	**0.039**	0.29 (0.09–0.94)		
Total dose (Gy)				
≤ 50				
> 50	0.752	0.85 (0.30–2.38)		
Total dose EQD2 (α/β = 3) (Gy)				
≤ 55				
> 55	0.103	0.42 (0.15–1.19)		
Overlap time of EGFR-TKI and TRT (days)				
≤ 20				
> 20	**0.008**	8.52 (1.75–41.41)	**0.011**	9.11 (1.67–49.65)
Duration of TRT (days)				
≤ 33				
> 33	**0.048**	3.05 (1.01–9.21)		
Ipsilateral lung V30 (%)				
≤ 34				
> 34	**0.015**	7.50 (1.48–38.08)	**0.036**	7.48 (1.14–49.14)
Timing of TRT was added				
TRT was added after the PD in the maintain of TKI				
TRT was added without PD in the maintain of TKI	0.631	1.32 (0.43–4.06)		

The bold text was some risk factor of RP, which were significantly correlated with RP and the *p*-value < 0.05

*EGFR-TKI* epidermal growth factor receptor tyrosine kinase inhibitor, *TRT* thoracic radiotherapy, *CFRT* conventional fractional radiotherapy, *SBRT* stereotactic body radiation therapy, *GTV* gross tumor volume, *PTV* plan tumor volume, *RP* radiation pneumonitis, *PD* progressive disease

#### Multivariate analysis

**Fig. 2:**
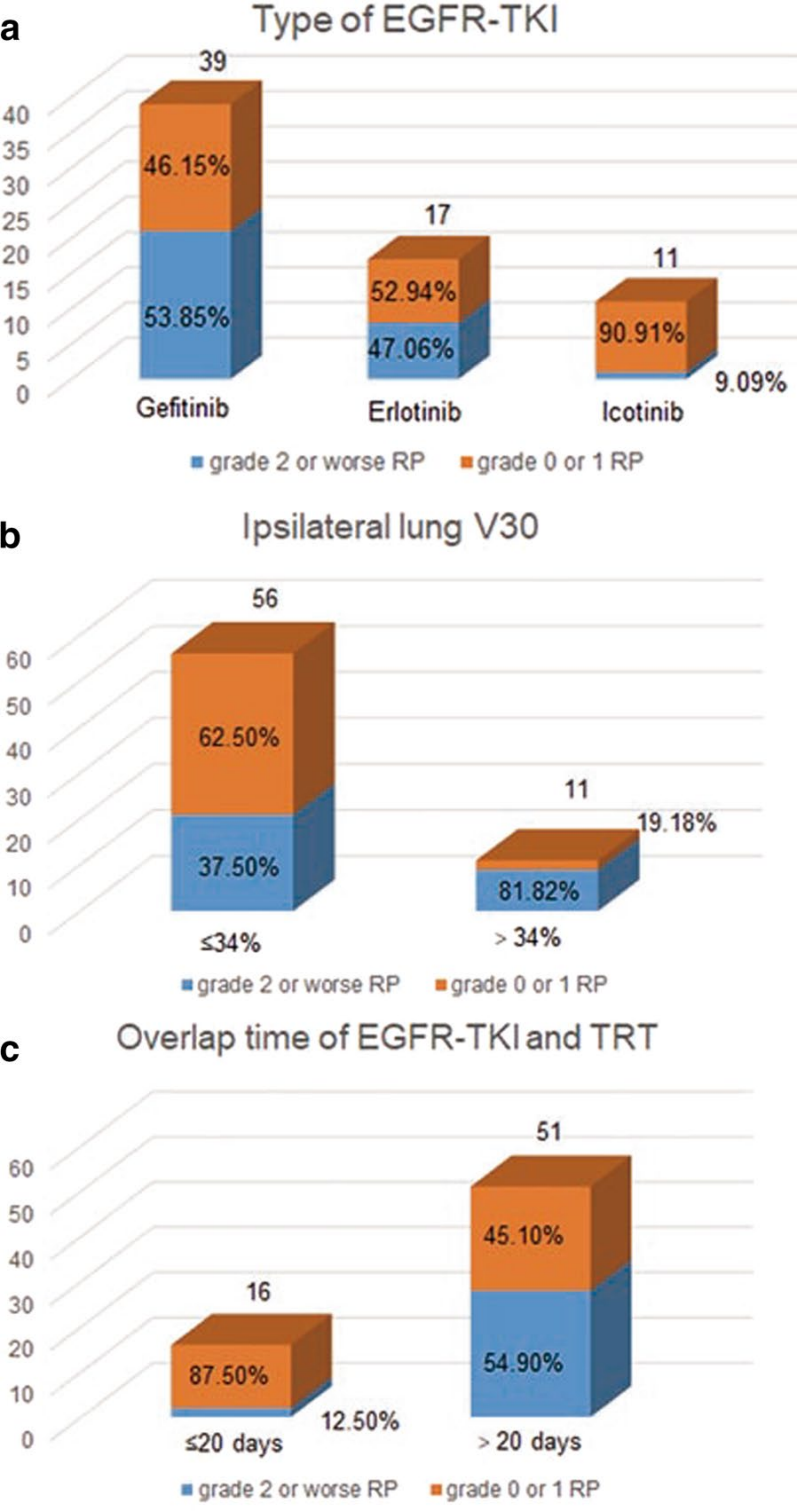
Rates of radiation pneumonitis by subgroup of **a** type of EGFR-TKI, **b** ipsilateral lung V30, and **c** overlap time of EGFR-TKI and thoracic radiotherapy

In consideration of the collinearity of some variates, the type of EGFR-TKI, PTV, dose per fraction, overlap time of first-generation EGFR-TKI and TRT, and ipsilateral lung V30 were included in multivariate analysis. The results are presented in Table 3. The type of EGFR-TKI, ipsilateral lung V30, and overlap time of first-generation EGFR-TKI and TRT were independent predictive factors of grade ≥ 2. Icotinib yielded a lower rate of grade ≥ 2 RP than did gefitinib (OR 0.075; 95% CI 0.007–0.78; *P* = 0.030). Ipsilateral lung V30 ≥ 34% (OR 7.48; 95% CI 1.14–49.14; *P* = 0.036) and an overlap time of > 20 days for first-generation EGFR-TKI and TRT (OR 9.11; 95% CI 1.67–49.65; *P* = 0.011) were significantly correlated with grade ≥ 2 RP. The rates of grade ≥ 2 by subgroup of type of EGFR-TKI, ipsilateral lung V30, and overlap time of first-generation EGFR-TKI and TRT are shown in Fig. 2. The results showed that compared with gefitinib and erlotinib, icotinib may be a protective factor for RP. Ipsilateral lung V30 ≤ 34% also yielded a lower rate of grade ≥ 2 RP than ipsilateral lung V30 > 34%. Further, a shorter overlap time of EGFR-TKI and TRT may help lower the risk of grade ≥ 2 RP.

## Discussion

RP is an important complication of simultaneous treatment with first-generation EGFR-TKI and TRT for EGFR-mutant NSCLC. However, its exact rate and risk factors in this population are unclear. In this study, 44.78% (30/67) of the patients simultaneously treated with first-generation EGFR-TKI and TRT developed grade ≥ 2 RP. In univariant analysis, a dose per fraction of ≥ 3 Gy was found to be a protective factor of grade ≥ 2 PR which is conflict with our knowledge, after correlation analysis, we found the dose per fraction was negative correlation with overlap time and PTV, so we think the result is only a statistical correlation and can be explained by a shorter overlap time and smaller PTV. Through multivariate analysis we found the EGFR-TKIs gefitinib and erlotinib, ipsilateral lung V30 > 34%, and overlap time of > 20 days for EGFR-TKI and thoracic radiotherapy increased the risk of grade ≥ 2 RP.

Combination treatment with first-generation EGFR-TKI and TRT is being increasingly used owing to its profound prognostic benefit [3, 5, 7]. However, RP has also been frequently reported in this modality [3, 8, 9]. Further, previous phase I clinical trials to explore the safety of simultaneous EGFR-TKI and TRT for unresectable untreated stage III NSCLC harboring EGFR mutations were terminated earlier owe to lung toxicity [11, 12]. Consistent with the findings reported by Zhuang et al. [13], the rate of grade ≥ 2 RP for patients treated with the combination of EGFR-TKI and TRT was higher than that for patients receiving concurrent chemoradiotherapy. Similar findings were reported by Zheng et al. and Chang et al. who reported that 20% (2/10) and 12% (3/25) of their patients developed grade ≥ 3 RP when treated with this modality [6, 14].

The high rate of grade ≥ 2 RP may be explained by some therapeutic mechanisms. First, as the target of EGFR-TKI, EGFR is primarily expressed in alveolar epithelial cells and is important for their proliferation and regeneration. EGFR inhibition suppresses the proliferation of alveolar epithelial cells and prevents their self-repair in response to radiation damage [15]. Second, erlotinib combined with radiation induces the accumulation of tumor cells in the G_1_ and G_2_-M phase, resulting in a reduction of them in the S phase. Erlotinib was also found play important roles in apoptosis induction, accelerated cellular repopulation, DNA damage repair, and enhanced radiosensitivity of tumor cells. All these can also increase radiation injury in normal lung tissue [16]. In addition, Harada et al. reported that gefitinib can upregulate important genes, including S100A8, S100A6, and stefinA3. These genes are known to be involved in neutrophil sequestration, acute inflammation, and airway remodeling [17]. Miyake et al. also reported that gefitinib can increase inflammatory cell infiltration and pro-inflammatory cytokine expression and augment pneumonitis [18]. However, further studies are still needed to elucidate the exact mechanism for the high rate of RP in combination treatment with first-generation EGFR-TKI and TRT.

Zhuang et al. reported that the lung dosimetric parameters and PTV are the predictive factors of RP for patients simultaneously treated with EGFR-TKI and TRT [19]. Consistent findings were observed in our study. In this study, we found that type of EGFR-TKI, ipsilateral lung V30, and overlap time of first-generation EGFR-TKI and TRT are independent predictive factors for grade 2 or worse RP. Patients treated with icotinib have a lower rate of grade ≥ 2 RP than gefitinib and erlotinib, which is consistent with previous reports that icotinib has a lower rate of adverse events than gefitinib and has the narrowest and safest toxicity spectrum [20, 21]. The broad therapeutic window and high selectivity towards the target EGFR of icotinib may be a reason for the safer toxicity spectrum [22]. Additionally, icotinib is metabolized by several enzymes and has a shorter half-life than other EGFR-TKIs, thus decreasing the accumulation of icotinib [23–25]. This result highlights that icotinib may be a safer choice for simultaneous treatment with TRT. The total lung V20 and MLD have been verified to be reliable biomarkers for predicting the risk of RP [26, 27]. In this study, we found that the ipsilateral lung V30 is the most powerful predictor of grade ≥ 2 RP in simultaneous treatment with first-generation EGFR-TKI and TRT. An ipsilateral lung V30 of > 34% was an independent risk factor. An overlap time of > 20 days for the first-generation EGFR-TKI and TRT was also an independent risk factor. This indicates that a shorter overlap time may be safer. Given that the type of EGFR-TKI and overlap time of EGFR-TKI and TRT were associated with the risk of grade ≥ 2 RP, the EGFR-TKI may be responsible for the high rate of grade ≥ 2 RP in this combination treatment.

Despite the related adverse effects, TRT should not be completely ruled out as it remains beneficial for patients who develop disease progression. Possible solutions to mitigate the adverse effects include performing low-dose radiotherapy, using icotinib, or suspending EGFR-TKI treatment during TRT for patients at high risk of RP.

## Conclusion

In conclusion, the frequency of grade ≥ 2 RP is higher in simultaneous EGFR-TKI and TRT treatment than that in concurrent chemoradiotherapy. Icotinib, ipsilateral lung V30 ≤ 34%, and shorter overlap time of ≤ 20 days for EGFR-TKI and thoracic radiotherapy were protective factors against the risk of RP in these patients. Caution should be exercised in the use of simultaneous TRT and EGFR-TKI, and the treatment strategies can be optimized to reduce the rate of RP for patients treat with simultaneous EGFR-TKI and thoracic radiotherapy.

## Data Availability

All data generated or analysed during this study are included in this published article.

## Abbreviations

CT: Computed tomography
EGFR-TKIs: Epidermal growth factor receptor-tyrosine kinase inhibitors
GTV: Gross tumor volume
LAT: Local ablation treatment
MLD: Mean lung dose
NSCLC: Non-small-cell cancer
PD: Progressive disease
RP: Radiation pneumonitis
ROC: Receiver operating characteristic
TRT: Thoracic radiotherapy
